# Clinical Image: Esophageal Compression Secondary to Diffuse Idiopathic Skeletal Hyperostosis

**DOI:** 10.7759/cureus.113221

**Published:** 2026-07-23

**Authors:** Alberto Lopez Menchero Mora, David Velasco Sanchez, Marco Aurelio Ramirez Huaranga, Rafael Morcillo Carratala, David Castro Corredor

**Affiliations:** 1 Rheumatology, Hospital General Universitario de Ciudad Real, Ciudad Real, ESP; 2 Radiology, Hospital General Universitario de Ciudad Real, Ciudad Real, ESP

**Keywords:** cervical, diffuse idiopathic skeletal hyperostosis, dysphagia, esophageal compression, osteophytes

## Abstract

Diffuse idiopathic skeletal hyperostosis (DISH) is a non-inflammatory disorder characterized by ossification of spinal ligaments and entheses. Although thoracic involvement is most common, cervical disease may rarely cause clinically significant esophageal compression and dysphagia. We present the case of a 73-year-old man with progressive dysphagia whose videofluoroscopic swallowing study, cervical computed tomography, and magnetic resonance imaging demonstrated prominent anterior cervical osteophytes causing marked pharyngoesophageal compression, consistent with DISH. This case highlights the importance of considering cervical DISH in the differential diagnosis of mechanical dysphagia in older adults and illustrates the value of cross-sectional imaging in establishing the diagnosis and guiding multidisciplinary management.

## Introduction

Diffuse idiopathic skeletal hyperostosis (DISH) is a systemic non-inflammatory condition characterized by ossification of spinal ligaments and excessive osteophyte formation, predominantly affecting the thoracic spine, although other spinal regions may also be involved [1]. Clinically, DISH is often asymptomatic; however, it can occasionally cause pain, stiffness, and functional impairment. Cervical involvement may lead to significant complications, including airway obstruction, dysphagia, or, in rare cases, vocal cord dysfunction [2].

The pathophysiology of DISH is complex, involving both mechanical and metabolic factors in the development of abnormal bone proliferation. Associations with advanced age, male sex, metabolic disorders, and diabetes mellitus have been documented [3,4]. Imaging studies, particularly computed tomography (CT) and magnetic resonance imaging (MRI), are essential for accurate diagnosis and for assessing the extent of osteophytic compression on the esophagus and airway [2,5].

Recent literature emphasizes the importance of early recognition of cervical DISH in patients presenting with unexplained dysphagia to prevent serious complications such as aspiration pneumonia and respiratory compromise [1,2]. In this report, we present the case of a 73-year-old male with progressive dysphagia, in whom endoscopy, CT, and MRI revealed prominent anterior cervical osteophytes consistent with DISH, causing marked posterior pharyngoesophageal compression. This case highlights the necessity of correlating clinical findings with imaging to ensure timely diagnosis and management.

## Case presentation

A 73-year-old man with a medical history of paroxysmal atrial fibrillation receiving anticoagulant therapy and hyperuricemia, treated with rivaroxaban, bisoprolol, and allopurinol, was referred to the Rheumatology Department for evaluation of solid-food dysphagia associated with choking episodes.

**Figure 1:**
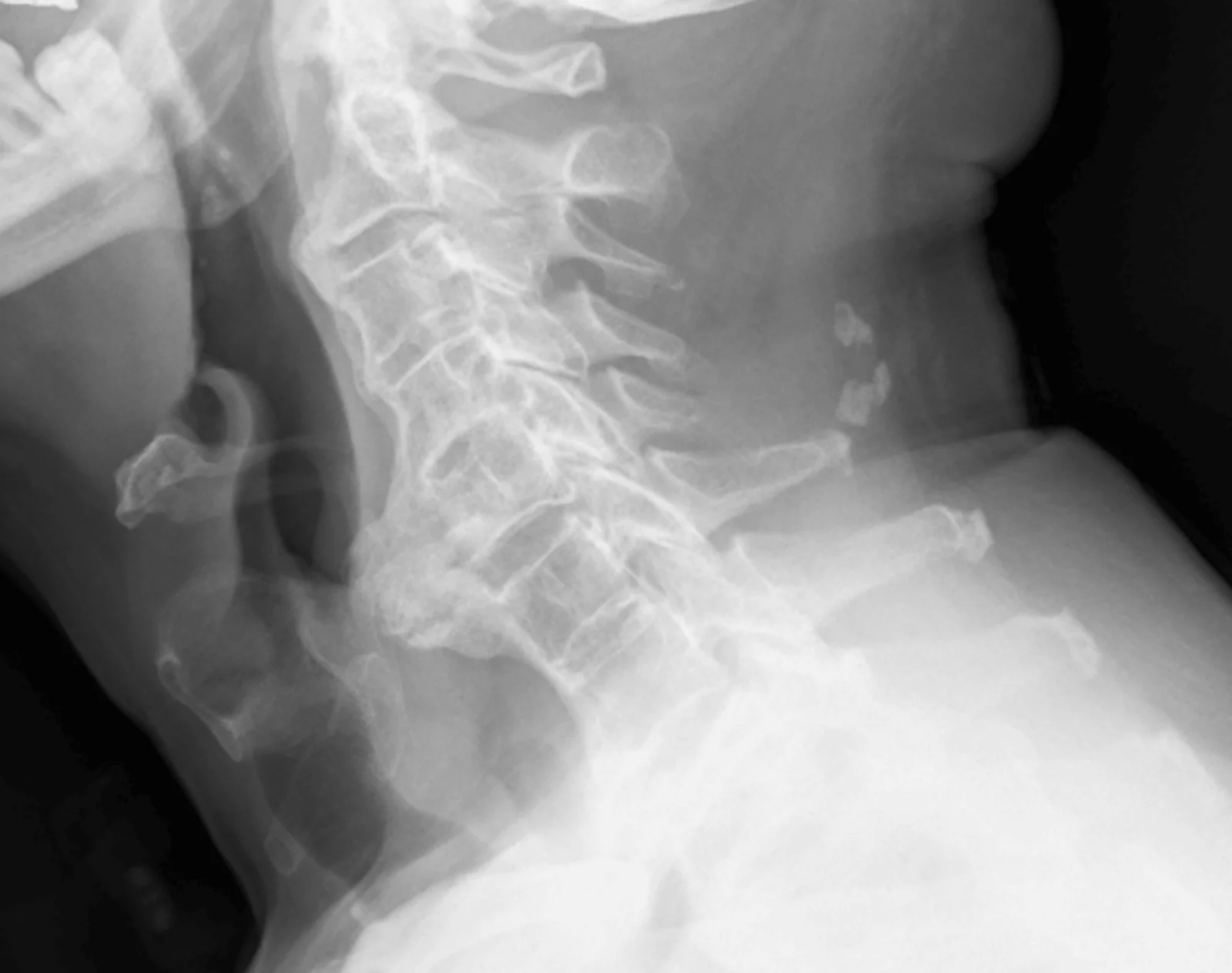
Lateral Cervical Spine Radiograph Lateral radiograph of the cervical spine demonstrating prominent, flowing anterior ossification along the anterolateral aspect of multiple contiguous vertebral bodies, consistent with ossification of the anterior longitudinal ligament. Intervertebral disc spaces are relatively preserved, and there is no radiographic evidence of significant apophyseal joint ankylosis.

Initial cervical radiographs demonstrated prominent anterior osteophyte formation involving the C4-C6 vertebral levels (Figure 1). The patient denied neck pain or dyspnea. Further evaluation included esophagogastroduodenoscopy (EGD) with videofluoroscopic swallowing study, followed by CT and MRI.

**Figure 2:**
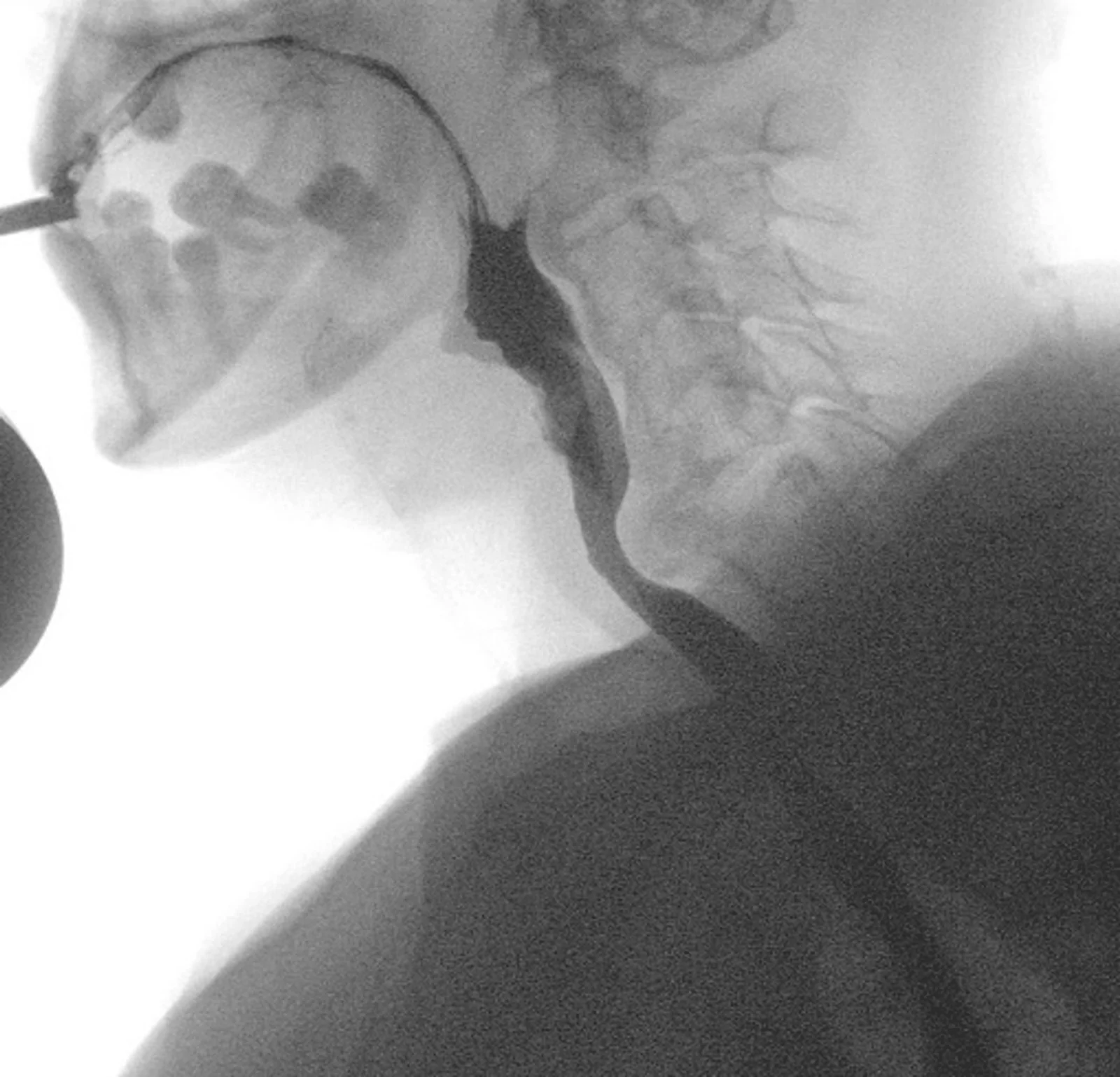
Esophagogastroduodenoscopy (EGD) EGD demonstrating marked posterior pharyngoesophageal compression, suggestive of extrinsic compression.

**Figure 3:**
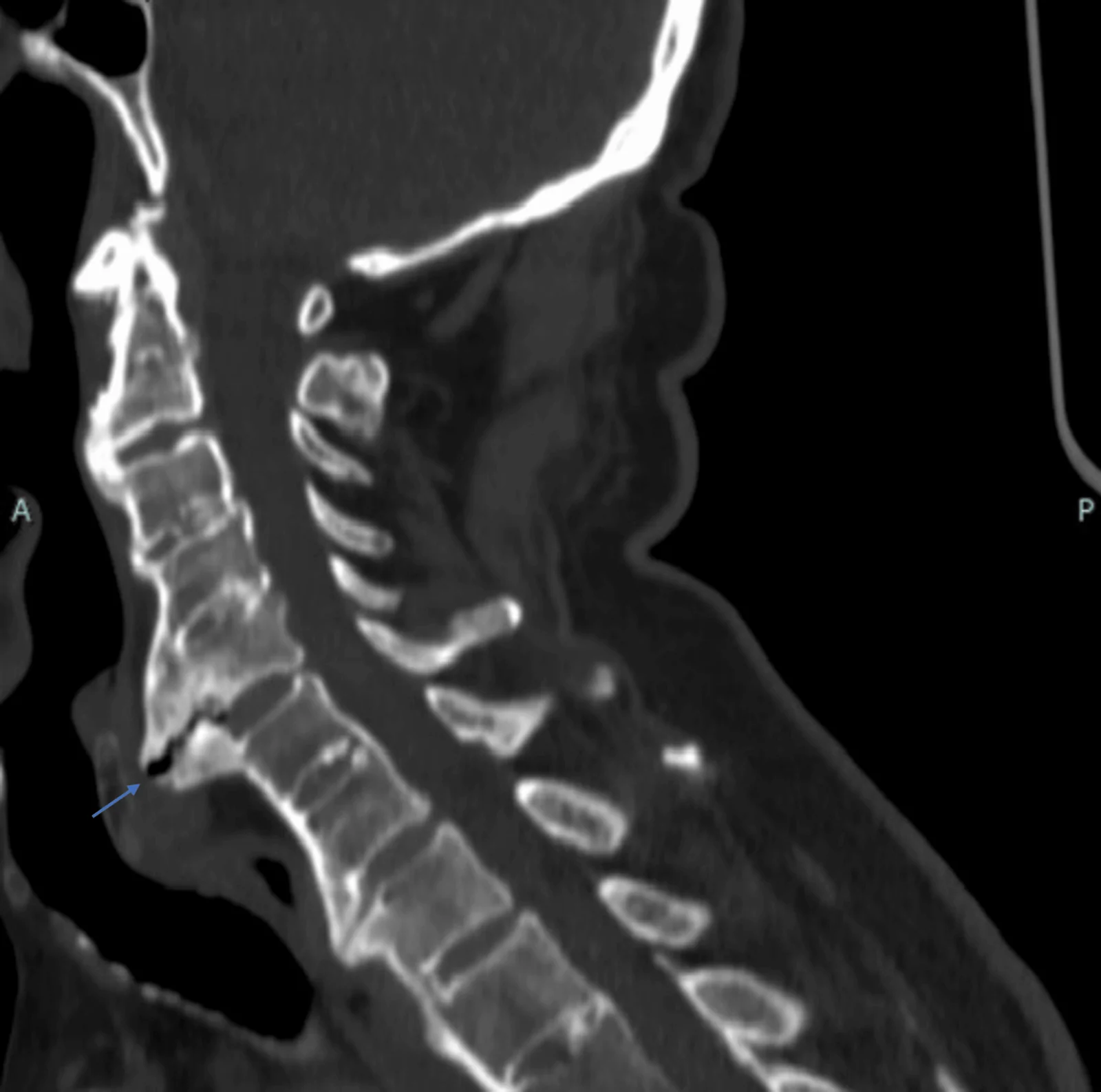
Computed Tomography (CT) Sagittal and axial cervical CT images showing prominent anterior marginal osteophytes, particularly at C5-C6, causing significant impression on the esophageal lumen (arrow). Findings are compatible with advanced diffuse idiopathic skeletal hyperostosis.

**Figure 4:**
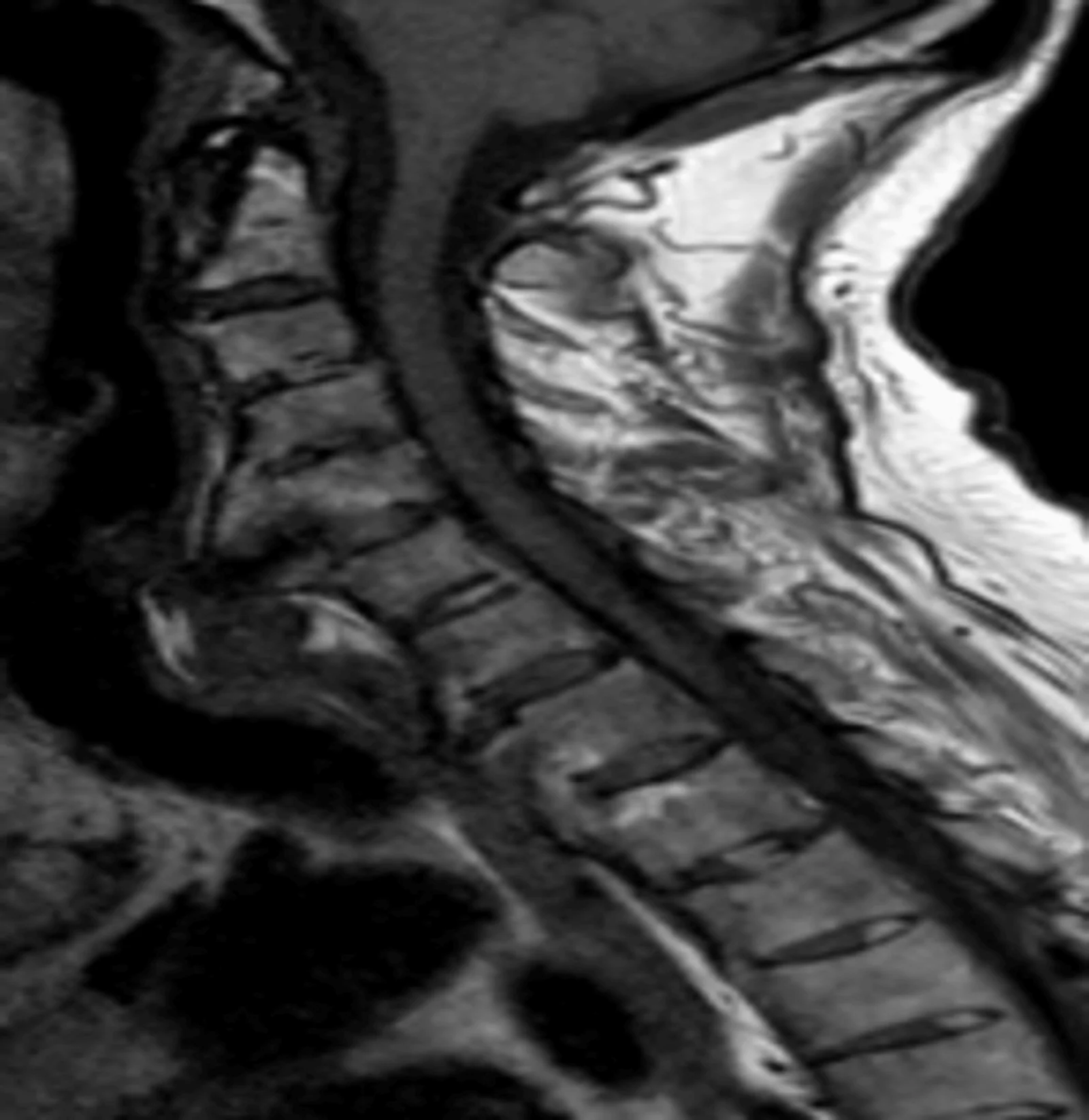
Magnetic Resonance Imaging (MRI) Sagittal cervical MRI demonstrating ossification of the anterior longitudinal ligament with bridging ossification at C3-C4 and C4-C5. Prominent right anterolateral marginal osteophytes are identified at C5-C6 and C7-T1, consistent with diffuse idiopathic skeletal hyperostosis (DISH).

Upper gastrointestinal endoscopy demonstrated marked posterior pharyngoesophageal compression (Figure 2). CT revealed prominent anterior marginal osteophytes, particularly at C5-C6, causing significant indentation of the esophageal lumen. These imaging findings were consistent with DISH (Figure 3). MRI demonstrated ossification of the anterior longitudinal ligament with bridging ossification across C3-C4 and C4-C5. Prominent right anterolateral anterior osteophytes were also identified at C5-C6 and C7-T1, further supporting the diagnosis of DISH (Figure 4).

The radiological findings, together with the clinical presentation, were consistent with DISH causing secondary esophageal compression. The imaging findings fulfilled the Resnick and Niwayama diagnostic criteria for DISH [6]. The patient was referred to the Department of Neurosurgery for further evaluation. Following multidisciplinary assessment, he was considered a suitable candidate for anterior cervical osteophytectomy with intraoperative tissue sampling.

## Discussion

Cervical involvement in DISH represents an underrecognized structural cause of dysphagia in elderly patients, despite its reported prevalence of up to 32% in geriatric cohorts [7]. The formation of exuberant anterior osteophytes may result in esophageal compression and compromise of the upper airway, leading to clinical manifestations that include progressive dysphagia, dysphonia, and aspiration [8].

The primary mechanism of dysphagia in this setting is related to direct esophageal displacement and compression by anterior cervical osteophytes, as well as reduced cervical mobility secondary to vertebral ankylosis, which impairs the biomechanics of effective swallowing [9]. In the present case, this mechanism was identified as the main contributor to esophageal symptoms. The typically anterior and symmetric pattern of ossification explains the compression observed on imaging studies and its clinical correlation [7].

CT plays a central role in diagnosis, as it allows precise assessment of the extent of ossification and its anatomical relationship with the esophagus and trachea, thereby facilitating the differential diagnosis from other structural causes of dysphagia [8,10]. In our case, correlation between clinical findings and CT and MRI was essential to establish the diagnosis according to the Resnick and Niwayama criteria while excluding other causes of dysphagia, including axial spondyloarthritis, degenerative cervical disease, and neoplasia [6].

Although initial management is usually conservative, surgical osteophytectomy may be considered in patients with significant dysphagia or respiratory compromise. Previous case series have reported symptomatic improvement in most patients following surgery [1].

This case underscores the importance of considering cervical DISH in the differential diagnosis of progressive dysphagia in older adults and highlights the value of clinicoradiological correlation to ensure appropriate management and prevent potentially serious complications.

## Conclusions

Cervical DISH is a relevant and often underrecognized structural cause of dysphagia in elderly patients. A high index of clinical suspicion is warranted in elderly patients presenting with unexplained dysphagia, particularly when anterior cervical osteophytosis is identified on imaging studies. Clinicoradiological correlation enables accurate diagnosis and appropriate management, preventing potentially serious complications. This case highlights cervical DISH as an important and treatable cause of progressive dysphagia, emphasizing the value of cross-sectional imaging for accurate diagnosis.
